# Density Functional Theory Study of Atomic Layer Deposition of Zinc Oxide on Graphene

**DOI:** 10.1186/s11671-015-1008-y

**Published:** 2015-07-22

**Authors:** Amgad Ahmed Ali, Abdul Manaf Hashim

**Affiliations:** Malaysia-Japan International Institute of Technology, Universiti Teknologi Malaysia, Jalan Sultan Yahya Petra, 54100 Kuala Lumpur, Malaysia

**Keywords:** Density functional theory, Graphene oxide, Atomic layer deposition, Zinc oxide, Acetylacetonate, Nanostructure

## Abstract

The dissociation of zinc ions (Zn^2+^) from vapor-phase zinc acetylacetonate, Zn(C_5_H_7_O_2_)_2_, or Zn(acac)_2_ and its adsorption onto graphene oxide via atomic layer deposition (ALD) were studied using a quantum mechanics approach. Density functional theory (DFT) was used to obtain an approximate solution to the Schrödinger equation. The graphene oxide cluster model was used to represent the surface of the graphene film after pre-oxidation. In this study, the geometries of reactants, transition states, and products were optimized using the B3LYB/6-31G** level of theory or higher. Furthermore, the relative energies of the various intermediates and products in the gas-phase radical mechanism were calculated at the B3LYP/6-311++G** and MP2/6-311 + G(2df,2p) levels of theory. Additionally, a molecular orbital (MO) analysis was performed for the products of the decomposition of the Zn(acac)_2_ complex to investigate the dissociation of Zn^2+^ and the subsequent adsorption of H atoms on the C_5_H_7_O_2_ cluster to form acetylacetonate enol. The reaction energies were calculated, and the reaction mechanism was accordingly proposed. A simulation of infrared (IR) properties was performed using the same approach to support the proposed mechanism via a complete explanation of bond forming and breaking during each reaction step.

## Background

Two-dimensional (2D) sheets of *sp*^2^-hybridized carbons known as graphene have attracted considerable attention because of their exceptional optical, electrical, chemical, and mechanical properties that impart graphene with the promising ability to develop next-generation functional nanomaterials for various applications [1–3]. To tailor graphene to targeted applications, considerable efforts have been made to control and modify the properties of graphene through various functionalization routes [4]. Furthermore, many studies have been conducted to develop semiconducting material/graphene hybrid structures using either vapor-phase [5–7] or liquid-phase techniques [8–11]. In the past few decades, zinc oxide (ZnO) nanostructures have been considered in many works for optoelectronic and photovoltaic device applications [9–11]. Recently, ZnO/graphene hybrid nanostructure was reported to have excellent potential for use in transparent flexible electrical and optical devices, including flexible photovoltaics, displays, and light emitters [10–15]. The vapor-phase deposition of ZnO using β-diketonates such as acetylacetonate as the Zn precursor was reported as a promising route to grow ZnO nanostructures [12, 14]. Most studies on ZnO/graphene hybrid structures have focused on their structural morphologies and electronic properties [8, 16], whereas few have paid attention to the reaction mechanisms of the semiconducting species at reaction sites on the graphene surface [17, 18]. To our knowledge, no study has reported the reaction mechanism for the vapor-phase deposition of ZnO on graphene utilizing acetylacetonate as a Zn source. In this article, we report the possible reaction mechanism for the deposition of ZnO on pre-oxidized graphene via the injection of vaporized zinc acetylacetonate in the presence of hydrogen.

## Methods

Until the early 1990s, quantum chemists used the ab initio Hartree–Fock (HF) approach along with second-order Møller-Plesset perturbation theory as starting points to solve Schrödinger’s equation [19]. Further calculations based on experimental data were carried out for the sake of accuracy through quadratic configuration interaction or coupled cluster theory in the case of small molecules [19, 20]. It is only possible to solve the Schrödinger equation for a one-electron system. Thus, in the late 1980s, density functional theory (DFT) coupled with local density corrected approximation (LDA) was developed as an alternative approximation method to derive reliable solutions to the Schrödinger equation for many-electron systems. In computational physics and chemistry, the HF method is one of the approximation methods that is used to determine the wave function and energy of a quantum many-body system in a stationary state. However, according to the HF approximation, electrons move *independently*, meaning that both the electron–electron repulsion energy and their total energy are overestimated [20, 21]. The limiting HF energy is therefore higher than the experimental energy. The electron correlation energy is the term used to describe the coupling or correlation of electron motions and is defined as the difference between the HF energy and the experimental energy [20, 22, 23].

To overcome the limitation of the HF approximation, Becke reported a work on density functional thermochemistry in 1993 in which he used DFT coupled with gradient-corrected exchange functional (B88) in conjunction with the Lee-Yang-Parr gradient-corrected correlation functionals (LYP) [19, 24]. Later on, Becke introduced the Becke three-parameter Lee-Yang-Parr (B3LYP) hybrid approach that can overcome the HF approximation limits [19, 24–26]. The B3LYP approach is based on the so-called free electron gas and can be described as a box of non-interacting electrons. This hybrid approach was used to construct the density functional approximations in the present study. Its solution leads to a functional form for a term that accounts for electron correlation. This term, which depends on electron density as well as the gradient of the density presented in Eq. (1), is thus added to the HF Hamiltonian:

1 $$ {E}_{xc}^{\mathrm{B}3\mathrm{L}\mathrm{Y}\mathrm{P}}={E}_x^{\mathrm{LDA}}+{a}_0\left({E}_x^{\mathrm{HF}}-{E}_x^{\mathrm{LDA}}\right)+{a}_x\left({E}_x^{\mathrm{GGA}}-{E}_x^{\mathrm{LDA}}\right)+{E}_c^{\mathrm{LDA}}{a}_c\left({E}_c^{\mathrm{GGA}}-{E}_c^{\mathrm{LDA}}\right) $$

where *E*_*x*_^GGA^ and *E*_*c*_^GGA^ are the generalized gradient approximations, and a_0_, a_*z*_, and a_*c*_ are correlation constants equal to 0.20, 0.72, and 0.81, respectively [19, 27, 28]. This procedure is referred to as a density functional model. Contrary to popular belief, B3LYP was not fitted to experimental data. The three parameters defining B3LYP have been taken without modification from Becke’s original fitting of the analogous B3PW91 functional to a set of atomization energies, ionization potentials, proton affinities, and total atomic energies [28, 29].

### Computational Details

The Spartan 14 quantum chemistry package (Wavefunction, USA) was used to perform all calculations in this study [30]. Equilibrium geometries were optimized by the B3LYP density functional method using the 6-311G** basis set; the developer of Spartan chose the Gaussian exponents for polarization functions to give the lowest energies for the modeled molecules. The polarization of the *s* orbitals on hydrogen atoms is crucial to accurately describe the bonding in acetylacetonate systems, particularly the hydrogen bonding. Furthermore, the 6-31G** basis set provides the *p*-type polarization functions for hydrogen. This can improve the total energy of the system along with the results for systems with large anions and can impose more flexibility [31]. Zn-containing structures were also optimized with larger basis sets and higher levels of theory [31].

All thermal correction energies were calculated using the 6-311G**, 6-311++G**, and 6-311++G(2df,2pd) (for Zn-containing reactions) basis sets. Calculations involving anions and absolute acidity (e.g., dipole moment calculations) require extra care when selecting the basis sets because extra electrons are weakly coupled to specific atoms or groups of atoms. Thus, the basis sets should provide diffuse *s-* and *p*-type functions on non-hydrogen atoms. This is usually designated by the “+” sign, as in 6-311++G**. The second “+” sign indicates that a diffuse function is added to hydrogen [32, 33]. To obtain more accurate energy calculations, single-point calculations were performed at the B3LYP/ 6-311G** optimized geometry using the B3LYP/6-311 + G**, MP2/6-311 + G**, B3LYP/6-311 + G(2df,2p), and MP2/6-311 + G(2df,2p) levels of theory.

## Results and Discussion

### Dissociation of Zn^2+^ from the Zn(acac)_2_ Complex

**Fig. 1:**
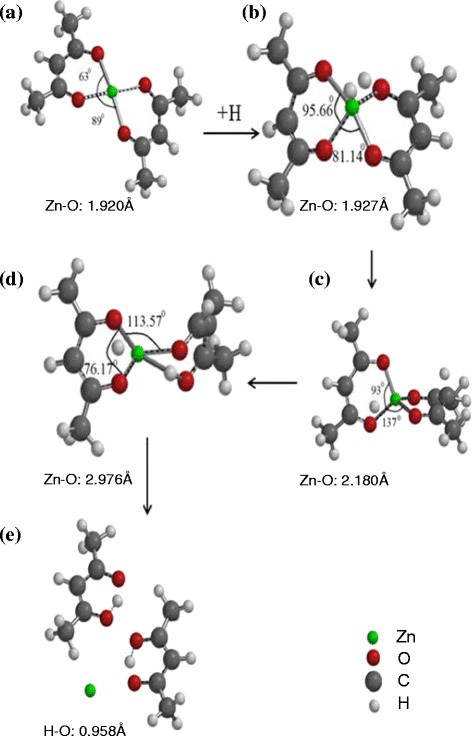
**a**–**e** Structures and geometries of transition states, intermediates, and products in the dissociation reaction

In this study, the geometries of reactants, transition states, and products were optimized at the B3LYP/6-31G** level of theory or higher. The optimized geometries of transition states, intermediates, and products during the dissociation reaction of Zn^2+^ from the Zn(acac)_2_ complex are depicted in Fig. 1. Figure 1a, b shows that rotation and stretching occurred in all the Zn–O bonds of the Zn(acac)_2_ complex upon the introduction of H atoms. The angle between Zn–O bonds in each of the chelates of the complex increased dramatically from 63.00° to 95.66°, indicating the adsorption of the H atoms towards the center of the complex. Figure 1c shows that both the torsion and stretching increase along the Zn–O bond, as indicated by the increase in the angle between the two chelates from 81.14° to 137.00°. The increase in the Zn–O bond length from 1.927 to 2.180 Å indicates the beginning of the tautomeric transformation of the complex. As shown in Fig. 1d, the Zn–O bond reaches a maximum length of 2.976 Å as the H atom moves closer to the center of the complex. Figure 1e shows the final step in which the ligand substitution is completed when a 0.958-Å-long hydrogen bond formed with the O atom and the Zn^2+^ was successfully extracted from the complex.

### Mechanism of the Dissociation Reaction

**Fig. 2:**
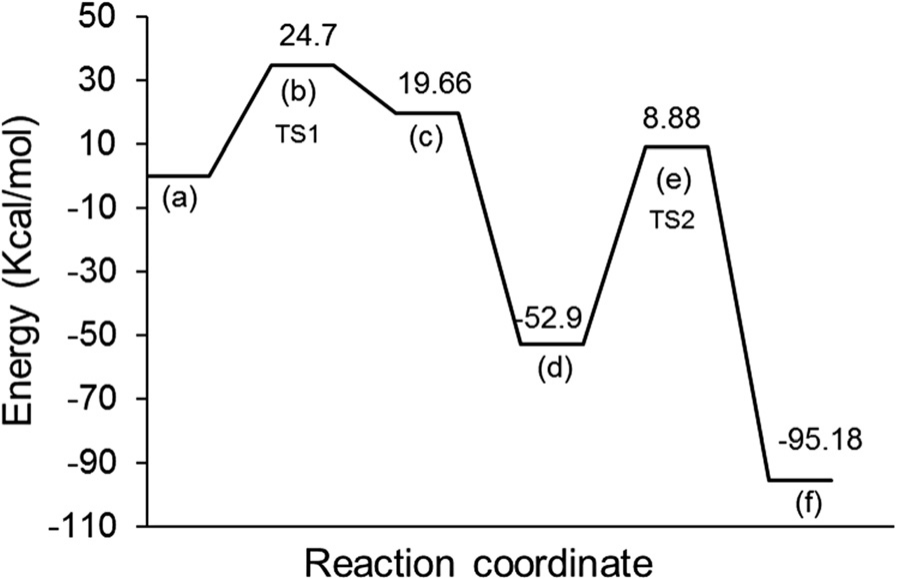
a–f Potential energy profile showing the relative energies for the dissociation reaction calculated at the B3LYP/6-311 + G(d,p) level of theory

The relative energies for the transition states, intermediate, and products in the gas-phase reaction mechanism were calculated at the B3LYP/6-311++G** and MP2/6-311 + G(2df,2p) levels of theory. The calculated energy data are depicted in the reaction coordinate pathway in Fig. 2. The reaction starts when two H atoms approach the Zn(acac)_2_ complex. H atoms are then adsorbed, as shown by step (a) in the reaction pathway in Fig. 2. The chemical reactions involve several distinct steps including two transition states (steps (b) and (e)) and one *intermediate* step (step (d)). The first transition state (TS1) occurs when secondary bonds are constructed between the 2H atoms and the O atoms. The relative energy for TS1 was calculated to be 24.70 kcal/mol (Fig. 2). The initial transition reaction leads to twisted and stretched Zn–O bonds at a calculated energy of 19.66 kcal/mol (step (c)). This reaction cycle appears to be endothermic because the energy of the products is higher than the energy of the reactants. As the reaction proceeds, Zn^2+^ dissociates due to the breaking of Zn–O bonds; consequently, the O–H bonds become stronger, and TS2 is formed (step (d), Fig. 2). The calculated energy barrier for the dissociation of Zn^2+^ was found to be 61.78 kcal/mol. The reaction is terminated when the O–H bond is formed at a calculated energy of −95.18 kcal/mol (step (e)). The overall dissociation reaction can be then summarized as shown in Eq. (2).

2 $$ \mathrm{Z}\mathrm{n}{\left({\mathrm{C}}_5{\mathrm{H}}_7{\mathrm{O}}_2\right)}_2+2{\mathrm{H}}_2\to {\mathrm{Zn}}^{2+}+2\mathrm{H}\left({\mathrm{C}}_5{\mathrm{H}}_7{\mathrm{O}}_2\right) $$

The reaction coordinate diagram shows that the initial transition state was obtained at a lower energy barrier (24.70 kcal/mol) than the final transition state (61.78 kcal/mol). Thus, TS1 is considered to be the rate-limiting step on which the overall reaction kinetics depend. The reaction follows a typical interchange substitution mechanism profile as the secondary bonding with the square planar complex of Zn(acac)_2_ were detected at the reaction intermediates. Because the association of H atoms to the square planar complex is the longest step in the pathway, it can be considered to be the actual rate-determining step of the overall reaction. Hence, the reaction mechanism can be classified as an interchange substitution mechanism that is associatively activated.

**Fig. 3:**
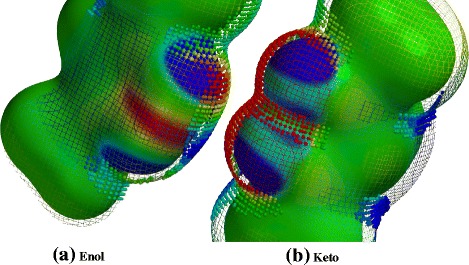
A merged electrostatic potential map (isosurface) and spin-density map (isocontours) for the acac enol tautomer (**a**) and keto tautomer (**b**)

To validate Eq. (2), the byproduct of the dissociation reaction must be studied. In fact, the acetylacetonate anions might react with H atoms in various rapid reactions; however, the enol and keto tautomers of the acetylacetonate compound are mostly expected to occur in the gas phase. To predict the favored tautomer that is produced as a byproduct of the dissociation reaction, the spin density, electrostatic potential distribution, bond orders, and bond lengths were computed. Figure 3 shows the spin-density map (isosurfaces) merged with electrostatic potential topology (isocontours) for both tautomers of the acetylacetonate molecule. These maps were generated by plotting both properties over an electron density surface. The electrostatic potential map aims to indicate the distribution and concentration of the charges over the entire molecule. Thus, the blue isosurfaces at the added H atom of the acac enol indicate a high concentration of negative (or less positive) charges in this area, with a maximum electrostatic potential of 634 kJ. On the other hand, the acac keto exhibited weaker electrostatic attractions. Spin-density maps were used to show the distribution of spins (angular momentum of unpaired electrons) all over the molecules. In Fig. 3a, the red isocontours at the added H of the enol indicate high spins attributed to the lone pairs of the oxygen atoms. The difference between the number of unpaired electrons and the total spin density at the H atoms is a measure of the degree of covalent character of the hydrogen–ligand bonds. In contrast, for the keto tautomer, almost no spin is observed around the added H atom, indicating the lack of lone pairs. These observations suggest that the enol tautomer to be more stable than the keto tautomer in the gas phase.

To provide a more quantitative analysis of the stability of the resulting acac compound, the atomic charges are calculated (Table 1). Two approaches (electrostatic and Mulliken) were used to calculate the atomic charges to overcome the sensitivity of the calculations to basis set. The relatively large negative charge on the central atoms (C2) of both the enol and keto tautomers is attributed to the back-donation of O atoms. The charge at the keto C2 is higher than that at the enol C2, indicating that more charge alternation occurs in the enol tautomer, inducing higher aromaticity in the enol molecule. This is emphasized by the bond orders presented in Table 2; the C1–O1 and C3–O2 bond orders are greater for the keto form than for the enol form. Furthermore, the C1–C2 and C2–C3 bonds of the enol tautomer are stronger than those of the keto tautomer, as indicated by the increased bond orders and decreased bond lengths. This bond strengthening may also stabilize the three centers of the π bonds of the enol ring, enhancing the aromaticity of the ring. The strengths of the O1–H1, O2–H1, and C2–H1 bonds provide a final measure of the stabilities of both tautomers. The large charge separation between the O1 and H1 (Table 1) indicates a highly polarized O1–H1 secondary; the same case is observed for the O2–H1 bond. Therefore, the electron pair may shift from H towards the O of the C=O bond, inducing a dipole moment that is positive at the H side and negative at the O side. The dipole moment was calculated to be 4.8 Debye and is oriented at 38.9° from the C–O axis. This strong dipole moment results in the partial shifting of the electron charge from hydrogen to oxygen. Thus, the positive–negative attraction between the charges generated by this shift strengthens the hydrogen bonds, preventing the further dissociation of the O–H portion of one acetylacetonate group. However, the charge separation between H1 and C2 is much smaller than between the enol O–H bonds and is therefore less polarized. These facts, along with the bond orders and lengths listed in Table 2, indicate that the O–H bond of the enol tautomer is stronger than the C2–H1 bond of the keto tautomer. In fact, the data shown in Tables 1 and 2 emphasize that the O–H bond in the enol tautomer is much stronger than the intermolecular hydrogen bonds. Hence, it is clear that the acac enol is the favored byproduct of the Zn^2+^ dissociation reaction.

**Table 1:** Computed atomic charges calculated for the keto and enol tautomers of acetylacetonate molecule

Atomic charge					
		Keto		Enol	
Atom		Electrostatic	Mulliken	Electrostatic	Mulliken
C1		0.809	0.307	0.992	0.370
C2		−0.845	−0.405	−0.815	−0.354
C3		0.846	0.328	0.872	0.305
C4		−0.750	−0.133	−0.798	−0.138
C5		−0.761	−0.138	−0.768	−0.123
O1		−0.306	−0.166	−0.441	−0.130
O2		−0.293	−0.135	−0.395	−0.144
H1		0.435	0.352	0.538	0.285
H2		0.267	0.136	0.263	0.116
H3		0.200	0.065	0.271	0.138
H4		0.246	0.114	0.252	0.126
H5		0.421	0.345	0.276	0.200
H6		0.210	0.071	0.250	0.105
H7		0.269	0.137	0.266	0.133
H8		0.249	0.122	0.241	0.111

**Table 2:** Computed bond orders and bond lengths for the keto and enol tautomers of the acetylacetonate molecule

	Keto form		Enol form	
Bond	Bond orders	Bond length	Bond orders	Bond length
C1–O1	1.455	1.380	1.260	1.401
C1–C2	1.238	1.417	1.287	1.39
C2–C3	1.204	1.408	1.478	1.433
C3–O2	1.473	1.382	1.128	1.328
C3–C5	1.011	1.463	1.015	1.465
C1–C4	1.005	1.460	1.019	1.466
C4–H2	0.943	1.101	0.971	1.101
C4–H3	0.961	1.109	0.934	1.106
C4–H4	0.970	1.101	0.945	1.104
C2–H1	0.457	1.484	–	–
C2–H5	0.470	1.461	0.939	1.103
C5–H6	0.962	1.100	0.972	1.108
C5–H7	0.939	1.106	0.938	1.106
C5–H8	0.968	1.100	0.955	1.105
O1–H1	–	–	0.444	0.958
O2–H1	–	–	0.449	0.958

### Simulation of IR Spectroscopy

**Fig. 4:**
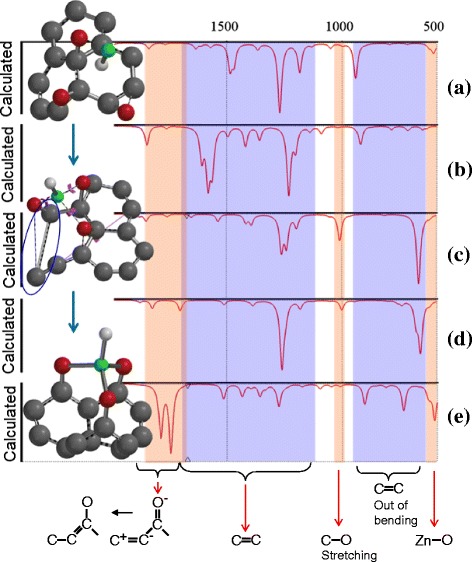
a–e IR data for the adsorption of Zn–H onto a graphene oxide matrix calculated using DFT and the corresponding optimized structures for various Zn^2+^ adsorption reaction steps

The infrared (IR) spectrum corresponding to the growth of ZnO nanostructures onto a layer of graphene was simulated using the DFT approach. The computational details were described earlier in this article. Because there is a hydrogen stream inside the reactor that is used to decompose Zn^2+^ from its complex, it is appropriate to assume that the released Zn^2+^ ion will be transported to the graphene oxide surface by hopping among the free H atoms. Thus, for the IR simulation, the Zn^2+^ is replaced with the Zn–H group. The simulation depicts the changes that happen during bonding between atoms after Zn–H was adsorbed at the oxygen sites on the surface of the graphene layer. For each reaction step, the IR peaks corresponding to every bond stretching, breaking, and forming were captured and plotted in Fig. 4 against the optimized geometry of the structure. Figure 4(a) shows that during the first step of the reaction, in which Zn–H was adsorbed, a C=C peak corresponding to vibration out of bending was observed in the range of 700–900 cm^−1^; this can be observed clearly in the corresponding structure geometry. This peak is attributed to the restricted rearrangement of C atoms in the graphene network to accommodate the approaching Zn–H. Accordingly, peaks corresponding to C–C stretching were also observed in the range of 900–1100 cm^−1^.

Figure 4(b) shows peaks at 1448–1560 cm^−1^, which are attributed to the conversion of the carbonyl group from C=C–C=O into the transient structure C^+^–C=C–O^+^. During this conversion, the symmetry about the carboxylic group increased, and the two peaks corresponding to the symmetric O–C–O bond of the anionic carboxylate group (at 1448 cm^−1^) and the asymmetric O–C–O bond (at 1560 cm^−1^) agreed with the reported spectra of carboxylate complexes (Table 3) [34, 35]. The spectral peak is still premature (low intensity), which indicates that the conversion process is starting, with the double bonds beginning to break into single bonds with secondary bonds. Furthermore, an interesting peak was observed at 1220–1400 cm^−1^ (Fig. 4(c)). This peak is attributed to the C=C stretching among the graphene C network, which is also observed in the corresponding optimized structure. Such stretching could take place to overcome the lattice mismatch between graphene and the ZnO crystal.

**Table 3:** The results of the IR simulations compared to published experimental results

Wave number	Simulation	Ref [34]	Ref [35]
900	C–C vibration out of bending		
Bands around 1000	C–C vibration out of bending		C–C vibrations
1100	C–C stretching and C–O bonds	C–O vibrations of the epoxy groups	Presence of νC–O bond
1220–1400	Attributed to the C=C stretching among the graphene C network	C–OH stretching, the C=C stretching	
1478–1560	Conversion of the carbonyl group from C=C–C=O into transient structure C^+^–C=C–O^+^		Peaks around 1478 due to the increase of O−C=O vibrations during the conversion of carbonyl group.
1630		Attributed to aromatic carbon double bonds	C=C bonds
1730	Complete transformation of the carbonyl group into C^+^–C=C–O^+^	Corresponding to the C=O stretching vibrations from carbonyl and carboxylic groups	Vibrations at 1700 indicating C=O bonds

As long as the reaction proceeds, the intensity of the previously stated peaks continues to change according to the continuous movement of the graphene oxide layer to accept the Zn–H group. The permanent bonds are constructed via the strong attachment of the Zn–H group to the oxygen sites. A remarkable peak was observed at 1730 cm^−1^ (Fig. 4(d)), indicating the complete transformation of the carbonyl group into C^+^–C=C–O^+^. In the next step of the simulation, a peak corresponding to Zn–O bond formation was detected (Fig. 4(e)) at 550 cm^−1^. In the corresponding geometry for the same simulation step, the Zn–H bonds have been constructed between the three surrounding O atoms. In fact, the Zn–O peak was observed in the early stages of the simulation with low transmittance intensity. These peaks could be captured as a result of the tendency of Zn^2+^ (as a Lewis acid) to form complexes dominated by highly directional covalent interactions with the oxygen networks before the Zn–O covalent bonds are finally formed.

## Conclusions

In this study, we have investigated the gas-phase reactions involved in the deposition of zinc and the adsorption of Zn^2+^ to the oxygen network to produce ZnO/graphene composites. The energies of reactants, transition states, and products were calculated, and a reaction mechanism for the dissociation of Zn^2+^ from its complex was proposed. The energy barrier for the dissociation of Zn^2+^ from the acetylacetonate complex was found to be 61.78 kcal/mol. Furthermore, the results of a molecular orbital study indicated the complete abstraction of Zn^2+^ from the acetylacetonate complex. The calculated IR results were in good agreement with experimental IR results reported in literature, validating the findings of the current study. The proposed route of growth involves a self-terminating reaction due to H capping at the end of the H–Zn–3O group. This supports the possibility of achieving atomic layer deposition (ALD) rather than chemical vapor deposition (CVD) while deposition occurs from the gas phase.
